# Comparative Genotyping of Malaysian Clinical Isolates of *Streptococcus pneumoniae* by Multilocus Sequence Typing and Multilocus Variable-Number Tandem Repeat Analysis

**DOI:** 10.21315/mjms-09-2024-677

**Published:** 2025-02-28

**Authors:** Nurul Asyikin Abdul Rahman, AbdulRahman Muthanna, Mohd Nasir Mohd Desa, Nurul Diana Dzaraly, Hazmin Hazman, Nurshahira Sulaiman, Norfazlina Mohamad, Maitasha Alia Meor Yahaya, Mazen M Jamil Al-Obaidi, Niazlin Mohd Taib, Siti Norbaya Masri

**Affiliations:** 1 Department of Biomedical Sciences, Faculty of Medicine and Health Sciences, Universiti Putra Malaysia, Serdang, Selangor, Malaysia; 2 School of Biology, Faculty of Applied Sciences, Universiti Teknologi MARA Negeri Sembilan, Kuala Pilah Campus, Kuala Pilah, Negeri Sembilan, Malaysia; 3 Department of Preclinical Sciences, Faculty of Dentistry, MAHSA University, Jenjarom, Selangor, Malaysia; 4 Biology Unit, Science Department, Rustaq College of Education, University of Technology and Applied Sciences, Rustaq, Oman; 5 Department of Medical Microbiology, Faculty of Medicine and Health Sciences, Universiti Putra Malaysia, Serdang, Selangor, Malaysia

**Keywords:** Streptococcus pneumoniae, sequence type (ST), multilocus sequence typing (MLST), multilocus variable-number tandem repeat analysis (MLVA), serotype, genetic lineages, Clonal Complex (CC)

## Abstract

**Background:**

*Streptococcus pneumoniae* (pneumococcus) is an opportunistic pathogen that causes severe upper and lower respiratory tract infections, leading to life-threatening diseases. This study aims to determine the genetic variation of serotypes among a collection of clinical *S. pneumoniae* isolates using multilocus sequence typing (MLST) and multilocus variable-number tandem repeat analysis (MLVA).

**Method:**

A total of 103 viable isolates were serotyped and subjected to MLVA; only those with discrete serotypes (*n* = 91) were subjected to MLST analysis.

**Results:**

The comparative phylogenetic analysis resulted in the segregation of clonal complexes (CC) and 36 singletons accordingly. The major clonal complex, MLST CC320 (*n* = 23; 25.3%), had a close association with the Taiwan^19F^-14 clone, consisting of ST236, ST271, and ST320. The second largest group, MLST CC9 (*n* = 12; 13.2%), had an association with the England^14^-9 clone, comprising ST7 and ST9. MLVA analysis demonstrated its ability to differentiate subgroups within CCs that share the same sequence type (ST) with distinct MLVA types (MTs).

**Conclusion:**

The integration of MLST and MLVA in this study serves as a model for pneumococcal surveillance indicating some well-known globally circulating clones that have been persisting at this study setting.

## Introduction

*Streptococcus pneumoniae* (pneumococcus) is an opportunistic gram-positive bacterium. It colonises the human nasopharyngeal region and causes both non-invasive and invasive pneumococcal disease (IPD) in children globally. IPDs result in more than a million annual fatalities, primarily impacting children under two years of age and elderly people, who are susceptible to infections such as septicaemia, meningitis, and otitis media (1–3).

Pneumococcal conjugate vaccines (PCVs) have significantly reduced the global burden of IPD such as pneumonia, and otitis media in children (4, 5). However, despite the reduction in disease burden associated with vaccine serotypes (VTs) after PCV implementation, IPD remains an important health problem due to the increase in non-vaccine serotypes (NVTs) (5–7). The extensive use of PCVs has led to “serotype replacement,” wherein NVTs have emerged and increased in prevalence as VTs being suppressed and decreased (8). As a deliberate response to the emergence of NVTs, new PCVs with extended serotype coverage were introduced. However, it is crucial to recognise the extensive variety of capsular polysaccharides present in *S. pneumoniae* and its capability to quickly adjust to certain pressures to escape the effect of vaccination (9, 10).

The genome of *S. pneumoniae* is complex, and modifications in the capsular polysaccharide (cps) loci can lead to variations in serotypes within a single strain. Pneumococci can alter their serotype through recombination events affecting the capsule polysaccharide synthesis (cps) locus. For vaccines to be effective in the long run, it is often necessary for low-virulence pneumococci to colonise. However, if there are frequent horizontal gene transfers within the bacterial population, it is possible for new variants with high virulence to emerge (11). Consequently, the widespread use of PCVs has led to the evolution and emergence of pneumococci in response to the vaccine usage pattern. This selective pressure has facilitated the emergence of new variant of *S. pneumoniae* strains, as well as an increase in the prevalence of capsule-switched strains (8). Therefore, the substitution of VT with NVT in the bacterial population is a significant concern because current pneumococcal vaccines provide protection only against the 24 known pneumococcal serotypes, while there are 101 identified serotypes (12). This capsular replacement phenomenon of VTs with NVTs and the expansion of NVTs is a natural adaptation and evolution leading to the emergence of increased pneumococcal virulence (11). Consequently, the elevated potential for infections caused by NVT, especially infections caused by highly antibiotic-resistant pneumococci, has become a substantial warning to societies and medical communities (13).

The selective pressure induced by PCV also leads to the emergence of clones associated with NVT and alterations in serotype profiles within established international epidemic clones. While clones typically belong to the same serotype, they may vary in their ability to cause invasive disease (14). Consequently, some genetic lineages within the same clonal group exhibit different serotypes and signalling potential serotype switches. This is concerning because it indicates potential changes in the virulence and serotype of the bacteria in the population (15).

Pneumococci that possess similar serotypes can exhibit genetic differences. Furthermore, certain serotypes may demonstrate a high degree of genetic relatedness despite being assigned different designations. This observation implies that the capsular serotype of pneumococci may play a more significant role than the genotype in determining their ability to induce invasive illness (15, 16). However, it is crucial to recognise that the pneumococcal genome has undergone continuous evolution to adapt to changing environment. This evolutionary process propels the spread of successful lineages, primarily associated with VT before the introduction of PCV. Their persistence may be sustained through the acquisition of NVT capsules. These dynamic mechanisms collectively shape the global population of circulating *S. pneumoniae* (7).

Genotyping based on nucleotide sequence variation of housekeeping genes, such as multilocus sequence typing (MLST), can estimate the relative contributions of recombinational exchanges and point mutations to the generation of variation within bacterial clones (17). MLST is a practical typing approach that can be used to investigate the population and evolutionary biology of *S. pneumoniae* and identify lineages, nomenclated as sequence type (ST) with high virulence and antibiotic resistance (18–20). The associated clonal complexes (CCs) consist of closely related pneumococcal STs that may spread globally, survive and persist for years. In addition, several single STs were found to belong to different serotypes, indicating a certain level of capsular switching (21).

Another typing approach that can identify genetic variations in pathogens and distinguish various fingerprint patterns of unrelated isolates is multilocus variable-number tandem repeat analysis (MLVA). MLVA is a DNA-based method that amplifies the variable-number of tandem repeats (VNTRs) in the microbial genome by detecting changes in the number of repeat units at numerous VNTR loci (19). Electrophoresis of PCR products may be used to assess size differences, and the number of repeat units may be calculated from the size of the detected amplicon (22, 23). Consequently, genotypes may be allocated based on the gain or deletion of tandem repeats, which provides a deeper understanding of the genetic relationships among pneumococcal strains (24). MLVA typing with 18 tandem markers was shown to have a good agreement with pulsed-field gel electrophoresis (PFGE) and MLST, suggesting that it could be used as an alternative or complementary approach that combines the efficiency of MLST with the accuracy of PFGE (25). Genotyping using MLST and MLVA will provide essential data on the diversity of isolates of similar and different serotypes and enable the investigation of the dissemination dynamics of international clones, their genetic relatedness, and the capacity to differentiate among strains with indistinguishable STs (23).

In this study, MLST and MLVA were used in combination with serotyping to characterise a collection of clinical pneumococcal isolates as a model for epidemiological investigation at the molecular level, with a particular focus on monitoring potential capsular gene switching at this study setting in Malaysia.

## Methods

### Bacterial Isolates

A total of 103 clinical isolates were viable in our culture collection. These isolates were collected in 2017–2019 from two tertiary hospitals located in major cities in Malaysia, from various isolation sites and patients of various ages (see Appendix 1). The isolates were reidentified for viability and purity following standard methods: colony morphology on Columbia agar with 5% sheep blood, gram-positive diplococci in the chain, a negative catalase test, bile solubility (Hardy Diagnostics, USA) and susceptibility test with ethylhydrocupreine or optochin (Liofilchem, Italy). All isolates were subjected to a standard protocol of sequencing, MLVA, and MLST respectively; the latter was performed on 91 clinical isolates with discrete serotypes only (non-typeable serotypes not included).

### Genomic DNA Extraction

The bacterial DNA was extracted from a few single colonies cultured on plates. DNA was extracted using the GeneAll ExgeneCell SV Mini Genomic Extraction Kit (GeneAll, South Korea). The colonies were placed in 100 μL of digestion buffer, heated to 100°C for 10 minutes, frozen at 20°C, thawed and centrifuged. The DNA-containing supernatant was stored at −20°C for future use.

### Molecular Identification of S. pneumoniae by Detection of The ply and lytA Genes

The *S. pneumoniae* virulence genes *(ply* and *lytA*) were amplified using PCR assays for verification (26, 27). PCRs mixed with a DNA template of *S. pneumoniae* ATCC 49619 served as a positive control, and the reaction mixture without the DNA template served as a negative control. All suspensions of the 25 μL PCR mixture were thoroughly mixed and run in a Bio-Rad MyCycler^TM^ Thermal Cycler (Bio-Rad, USA).

### Sequetyping Using The cpsB Single-gene Primer

Sequetyping was performed using primers designed by Leung et al. (28). The reaction mixtures contained 12.5 μl of MyTaq^TM^ Red Mix Bioline (Meridien, Biosciences, USA), 0.8 μl of forward primer (cps1), 0.8 μl of reverse primer, and 3 μl of genomic DNA, which made up a final volume of 25 μl with nuclease-free distilled water. The reaction cycle consisted of an initial denaturation step at 95°C for 5 minutes, followed by 30 amplification cycles of denaturation at 95°C for 30 seconds, annealing at 65°C for 30 seconds, extension at 72°C for 1 minute, and a final extension at 72°C for 5 minutes.

### Multilocus Sequence Typing

MLST analysis was conducted by amplifying the seven housekeeping genes (*aroE*, *gdh*, *gki*, *recP*, *spi*, *xpt*, and *ddl*) through PCR, employing the primers as specified at https://pubmlst.org/organisms/streptococcus-pneumoniae/primers (17). The internal fragments of these seven housekeeping genes, ranging from approximately 405 to 486 base pairs, were amplified. The reaction mixtures contained 50 μl of PCR mixture. The reaction cycle consisted of an initial denaturation step at 95°C for 1 minute, followed by 30 amplification cycles of denaturation at 95°C for 1 minute, annealing at 55°C for 15 seconds, extension at 72°C for 10 seconds, and a final extension at 72°C for 5 minutes.

To verify the sizes of the PCR products, 1.7% agarose gel electrophoresis with GelRed^®^ (Biotium, USA) was performed at 90 V for 60 minutes. All PCR products were subsequently subjected to Sanger sequencing at a commercial facility (Apical Scientific Sdn. Bhd. Malaysia). The sequences of the seven housekeeping genes were obtained and processed by trimming and aligning them using Molecular Evolutionary Genetics Analysis version 11 (MEGA 11) software, and referencing known alleles on the MLST database website (http://pubmlst.org/) to determine the allelic numbers and STs. New alleles and STs were verified through submission to the MLST curator. Additionally, clonal complex (CC) analysis was conducted using PHYLOViZ software, which is accessible at http://www.phyloviz.net (29).

### Multilocus Variable-number Tandem Repeat Analysis

MLVA was conducted involving the amplification of 18 VNTR loci (Spneu 15 to Spneu 42) using primers previously described by Koeck et al. (24). The PCRs were carried out in 25 μL final volume reaction mixtures. PCR amplification was performed using a Bio-Rad MyCycler^TM^ Thermal Cycler (Bio-Rad, USA). The sizes of the PCR products were checked using 2% agarose gel electrophoresis and staining with GelRed^®^ (Biotium, USA) at 80 V for 90 minutes. The allelic profile of each *S. pneumoniae* strain was determined based on the observed allele sizes from the electrophoresis gel image. Subsequent analysis of the MLVA numerical profiles was performed using PHYLOViZ, which is accessible at http://www.phyloviz.net (29). The Simpson’s index of diversity (SID) was used to compare the discriminatory power among the 18 VNTR loci. The diversity index (DI) was calculated to gauge the variation in the number of repeats at each locus, with values ranging from 0.0 (indicative of no diversity) to 1.0 (indicative of complete diversity). Thus, 7 VNTR loci were selected based on the combination with the highest discriminatory power for studying the population structure of *S. pneumoniae*. To facilitate systematic classification, MLVA types (MTs) and their respective CCs were designated using an in-house nomenclature. The MTs were assigned based on the allelic profile of the seven VNTR loci, with simple labelling ranging from MT1 to MT85. Using the unweighted pair group method with arithmetic mean (UPGMA) dendrogram analysis, a phylogenetic analysis was conducted on the 85 distinct MTs (repetitive MTs were excluded) using PHYLOViZ 2.0 software. The MLVA CCs were established when MT exhibited a minimum of 70% similarity, encompassing at least five identical loci out of the total seven loci analysed (25).

## Results

### Characteristics and Identification of S. pneumoniae

All 103 clinical isolates phenotypically demonstrated characteristic features of *S. pneumoniae* with the presence of the *lytA* and *ply* genes. These results indicate the viability and identity of the bacterium after a long-frozen state of preservation.

### Sequetyping Using The cpsB Single-gene Primer

All 103 isolates produced a 1061 bp band when amplified using the *cpB* single-gene primer. Upon sequencing and homology searches, 16 different serotypes were determined, and two isolates were undetermined and reported as non-typeable (NT). The most prevalent was serotype 19F (*n* = 22; 21.4%), followed by 19A (*n* = 13; 12.6%), 14 (*n* = 13; 12.9%), 23F (*n* = 12; 11.7%), 6A (*n* = 11; 10.7%), 6B (*n* = 10; 9.7%), 11A (*n* = 5; 4.9%), 3 (*n* = 3; 2.9%), 1 (*n* = 2; 1.9%), 4 (*n* = 2; 1.9%), 15C (*n* = 2; 1.9%), 35B (*n* = 2; 1.9%), 13 (*n* = 1; 1%), 15A (*n* = 1; 1%), 23A (*n* = 1; 1%, 34 (*n* = 1; 1%), and NT (*n* = 2, 1.9%).

### Multilocus Sequence Typing Analysis

**Figure 1:**
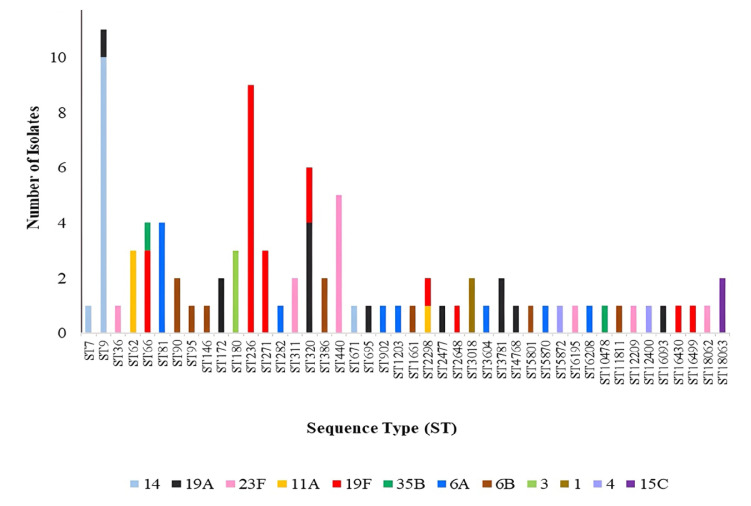
Distribution of STs of the clinical isolates with their corresponding serotypes

The seven targeted housekeeping genes were amplified in 91 isolates with discrete serotypes, revealing a wide range of STs among the pneumococcal isolates (Figure 1). The ST9 variant had the highest frequency (*n* = 11; 12.1%), within serotype 14 and one serotype, 19A. Subsequently, ST236 (*n* = 9; 9.9%) had a frequent association with serotype 19F, while ST320 (*n* = 6; 6.6%) displayed a common distribution across serotypes 19A and 19F. Moreover, ST 440 (*n* = 5; 5.5%) was associated with serotype 23F, ST66 (*n* = 4; 4.4%) was associated with two serotypes (19F and 35B), and ST81 (*n* = 4; 4.4%) was associated with serotype 6A. ST62, ST180, and ST271 were associated with a considerable number of clinical isolates (*n* = 3; 3.3%). Conversely, the remaining STs displayed a lower frequency of occurrence, with only one or two isolates observed for each of these STs. Among the various serotypes examined, both serotype 19A and serotype 19F displayed the greatest number of STs, each with eight STs. Serotype 19A exhibited associations with ST9, ST172, ST320, ST695, ST2477, ST3781, ST4768, and ST16093. Conversely, serotype 19F was linked to ST66, ST236, ST271, ST320, ST2298, ST2648, ST16430, and ST16499. Additionally, serotype 6A was found to be associated with ST81, ST282, ST902, ST1203, ST3604, ST5870, and ST6208. In contrast, serotype 6B was associated with ST90, ST95, ST146, ST386, ST1661, ST5801, and ST11811. Notably, serotype 3 was specifically associated with ST180, while serotype 1 was associated with ST3018, and serotype 15C was associated with ST18063. Hence, several STs were associated with specific serotypes, while others exhibited serotype heterogeneity.

The results revealed the presence of two new novel STs, ST18062 (*n* = 1) and ST18063 (*n* = 2). The latter demonstrated a relationship with serotype 15C, whereas ST18062 exhibited an association with serotype 23F. PHYLOViZ analysis using the MLST seven housekeeping gene sequences categorised the 91 isolates into 7 CCs and 22 singletons from 44 different STs, as shown in Table 1.

### Multilocus Variable-number Tandem Repeat Analysis

**Figure 2:**
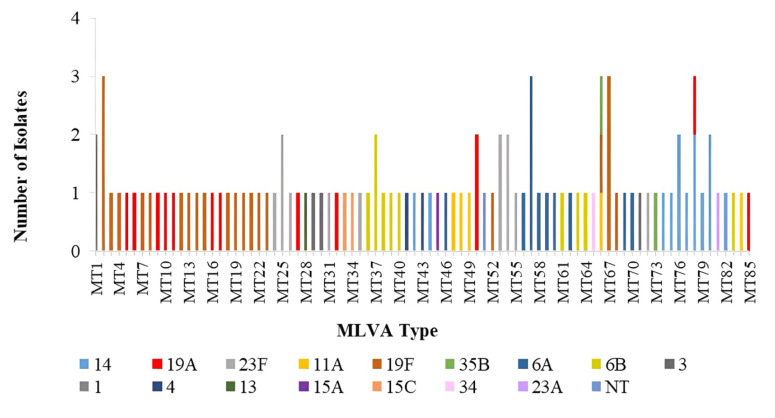
The Distribution of the clinical isolates with their MTs and associated serotypes

The analysis of the combinations of seven VNTR loci for the 103 isolates revealed a total of 85 distinct MLVA types (MTs). Figure 2 shows the distribution of these MTs and their corresponding serotypes.

Three clinical isolates are included within the respective MTs, namely, MT2, MT57, MT66, MT67, and MT68. Specifically, MT2 and MT67 are associated with serotype 19F, while MT57 is linked to serotype 6A. In contrast, MT66 exhibits a combination of multiple serotypes; 11A, 19F, and 35B. Moreover, MT78 is associated with serotypes 14 and 19A. Serotype 19F had the highest MT (*n* = 19; 18.4%), followed by serotype 19A (*n* = 12; 11.7%) and subsequently serotype 14 (*n* = 10; 9.7%).

**Figure 3:**
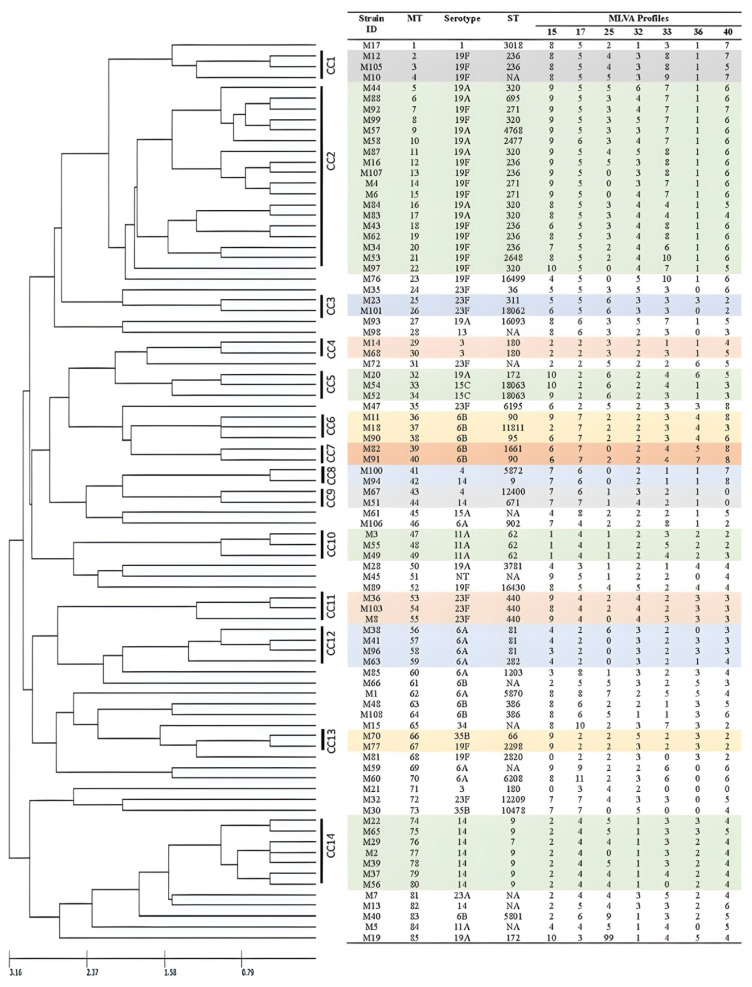
MLVA dendrogram deduced from Cluster Analysis of the 103 clinical isolates using the UPGMA Method Note: Each clonal cluster is distinguished by different colours, while singletons are depicted in white. CC = clonal complex; ST = sequence type; NA = data not available

The pneumococcal population structure is represented by MTs, which reveals the existence of 14 MLVA CCs, along with 29 isolates identified as singletons. Figure 3 displays the MLVA dendrogram, which effectively illustrates the clustering of CCs and singletons to demonstrate the relatedness of the clinical isolates.

### MLST Phylogenetic Analysis vs MLVA Types, Serotype (Sequetyping) and Demographic Data

The phylogenetic tree generated using a maximum likelihood analysis of the 91 clinical *S. pneumoniae* isolates demonstrated a bootstrap confidence interval value of 100% at all branching points. The MLST based phylogenetic tree, that included the global reference sequence, resulted in the segregation of the seven CCs and 36 singletons. To enhance the clarity of the presentation of the clinical isolation data and subsequent discussion, the analysis was organised into distinct segments based on the major MLST CCs. Each specific MLST CC, including the four major CCs (CC320, CC9, CC66, CC81) and the minor CCs (CC90, CC311, and CC146), was analysed alongside serotypes, MLVA types (MTs), sequence types (ST), isolation sites, isolation years, and patient ages, all further supported by the MLVA CC for a comprehensive analysis, as illustrated in Appendix 1. The observed clades of each specific MLST CC revealed a well-defined pattern of segregation with respect to the STs.

Phylogenetic tree analysis of MLST CC320 revealed a clear segregation into four clades, consisting of serotypes 19A and 19F. Furthermore, within MLST CC320, the clinical isolates were further grouped into CC1, CC2, and singleton categories based on the MLVA CC. This MLST CC320 also displayed invasive potential, as seen by the isolation of clinical isolates from blood samples encompassing a diverse range of age groups, ranging from early young children to elderly individuals.

Moreover, the MLST CC9 represented the second largest CC, revealing a single clade with two distinct subclades. The segregation within the subclades is predicated on the STs (ST7 and ST9). Concurrently, the MLVA CC analysis aligns with these phylogenetic findings, as most of the clinical isolates are clustered into MLVA CC14, with only one clinical isolate falling under CC8. Furthermore, MLST CC9 predominantly comprises serotype 14 clinical isolates, with the exception of a single clinical isolate belonging to serotype 19A. The clinical isolate distribution suggests a combination of invasive and non-invasive characteristics based on the various isolation sites. Notably, the age distribution within this clonal complex is highly diverse, encompassing individuals as young as 2 months to as old as 71 years.

MLST CC81 was grouped into one clade with three different subclades. The subclades were differentiated based on the respective STs (ST81, ST3604, and ST282). All the serotypes observed within this MLST CC81 belonged to serotype 6A. In terms of MLVA CC, the clinical isolates consistently clustered into CC12, with only one clinical isolate identified as a singleton. Notably, four different MTs were observed, namely, MT56, MT57, MT58, and MT59. MLST CC81 exhibited a remarkable diversity with respect to the isolation sites, encompassing both invasive sites involving blood and non-invasive sites involving sputum. The age distribution ranged from young children to elderly individuals between 2018 and 2019.

MLST CC66 comprises six clinical isolates organised into a single clade featuring two distinct subclades based on their respective STs (ST2298 and ST66). Within this group, a diverse combination of serotypes was observed, including serotypes 11A, 19F, and 35B. Additionally, MLVA CC13 was identified within MLST CC66, with only two distinct MTs, namely, MT66 and MT67. The age distribution within this CC demonstrated notable diversity, ranging from as young as 8 months to as old as 59 years. Nevertheless, it is notable that all the samples were collected during the year 2018. These isolates originated from both invasive sites and non-invasive sites.

MLST CC90 forms a single clade, which is further subdivided into three distinct subclades, each characterised by ST, namely, ST1661, ST95, and ST90. This MLST CC90 is exclusively associated with serotype 6B. Moreover, the MLVA CC clustered into two major groups, represented as MLVA CC6 and MLVA CC7. In terms of origins, the isolates were predominantly derived from non-invasive samples collected between 2017 and 2019. Regarding age distribution, the clinical isolates cover a wide age range, ranging from 2 years to 48 years.

MLST CC311 formed a single clade, which was subsequently divided into two distinct subclades based on the STs, namely, ST18062 (a novel ST) and ST311. These clinical isolates were exclusively associated with serotype 23F. MLVA CC3 was identified and exhibited two MTs, MT25 and MT26. The clinical isolates were collected in 2017 and 2018. Notably, the clinical isolates were obtained from both invasive and non-invasive cultures. The age range of individuals from whom the clinical isolates were obtained varied from 21 years to 59 years.

MLST CC146 comprises only two clinical isolates, which have been classified into a single clade further subdivided into two subclades based on their respective STs, namely, ST11811 and ST146. MLVA revealed their association with the MLVA CC6, and interestingly, both clinical isolates shared the same MT (MT37), indicating an identical allelic profile. The isolates clustered within MLST CC146 all shared the same isolation site, specifically blood samples collected in the year 2018. However, the age of individuals from whom the clinical isolates were obtained differed, with one sample being from a 4-year-old individual and the other from a 55-year-old individual.

Moreover, the MLST phylogenetic tree revealed the presence of 35 singletons, which are listed in Table 2. Most of the clinical isolates were categorised as singletons in both the MLST and MLVA analyses. However, among these clinical isolates, 17 were initially classified as MLST singletons but were subsequently classified as MLVA CCs. Three MLST singletons, namely, M20, M52, and M54, were further classified within MLVA CC5. Among these clinical isolates, two exhibited serotypes 15C and ST18063 (a newly assigned ST), while the third displayed ST172 and serotype 19A. Notably, each of these clinical isolates featured dissimilar MTs, with strain M20 categorised as MT32, M52 as MT34, and M54 as MT33. These clinical isolates were collected in 2018 and 2019 and originated from individuals of various ages: 1 month, 4 months, and 18 years.

Another interesting discovery pertains to a cluster of five MLST singletons, namely, M8, M9, M36, M103, and M104, all of which exhibit the common characteristics of belonging to ST440 and serotype 23F. This cluster was further classified as MLVA CC11, comprising three different MTs, namely, MT53, MT54, and MT55. These clinical isolates primarily originated from non-invasive sites, including sputum and eye swabs. The age range of individuals from whom the clinical isolates were obtained varied from 26 years to 63 years.

Three MLST singletons, namely, M55, M49, and M3, exhibit the common characteristics of serotypes 11A and ST62. These clinical isolates were subsequently grouped into MLVA CC10, which encompasses three distinct MTs, namely, MT47, MT48, and MT49. These clinical isolates were obtained during the years 2017 and 2019. Notably, the clinical isolates were collected from individuals of varying ages (32, 43, and 75 years) from various sources, including blood, sputum, and the eye.

One MLST singleton, denoted M88, was subsequently grouped into MLVA CC2. Similarly, another MLST singleton, labelled M100, was further clustered into MLVA CC8. Additionally, two different MLST singletons, M14 and M68, were subsequently categorised into MLVA CC4. Finally, two additional MLST singletons, M51 and M67, were further assigned to MLVA CC9. These clinical isolates are characterised by varying STs, which include ST12400, ST671, ST695, and ST5872, along with different serotypes encompassing serotypes 4, 14, and 19A. Notably, the two clinical isolates within MLVA CC4 share the same serotype 3 and exhibit the same ST (ST180). Eighteen additional MLST singletons are also classified as MLVA singletons, representing a range of sequence types (STs), allelic profiles (MTs), and serotypes.

## Discussion

Population genetics’ studies have significantly improved our understanding of the biology and epidemiology of important bacteria and fostered strategies for the management of global pathogens, including pneumococcus (30). MLST is a genotypic analysis that describes the molecular characteristics and genetic lineage of a bacterial population by sequencing the internal fragments of seven housekeeping genes (17). The fundamental advantage of MLST is the unambiguous nature of the sequence data, which facilitates a direct comparison of STs with specific serotypes with those archived within the central online database. Thus, the portability of MLST renders it essential for monitoring the geographical spread of successful internationally disseminated clones recognised by the Pneumococcal Molecular Epidemiology Network (PMEN) (18).

MLVA is a DNA-based molecular typing method that records the size of polymorphisms in VNTR loci amplified through stringent PCR protocols (31). VNTRs represent some of the most diverse genomic loci in bacterial populations (32). The variation generated at VNTR loci provides a high level of subtyping discriminatory power, making VNTR extremely useful molecular epidemiological markers (22). In contrast, MLVA is more suitable for short-term epidemiological changes and localised outbreaks (24, 33).

It was previously reported that there was considerable concordance between MLST and MLVA, but some of the isolates grouped by MLST could be further distinguished by MLVA (34). In parallel, the findings in the current study revealed a similar pattern of results, particularly for MLST CC320, MLST CC9, MLST CC81 and MLST CC90. Additionally, among the studied clinical isolates, 17 that were initially identified as MLST singletons were subsequently clustered into seven distinct MLVA CCs. This reclassification event was particularly obvious in the case of five clinical isolates belonging to ST440 and associated with serotype 23F, which were subsequently grouped within MLVA CC11. Moreover, three clinical isolates, identified as ST62 and affiliated with serotype 11A, underwent further differentiation and were ultimately allocated to MLVA CC10. Thus, it is apparent that MLVA can discriminate relevant subgroups among strains belonging to the same ST and offer the possibility to deduce the ST from the MT (23). MLST CC and MLVA CC were highly similar among MLST CC66, MLST CC311 and MLST CC146, suggesting the potential application of MLVA for the reliable identification of pneumococcal clones, as also reported by Costa et al. (25). Although these two methods measure different evolutionary mechanisms at multiple genomic loci, similar clustering was obtained, probably due to the infrequency of recombination (35). However, the ability of MLVA to precisely detect and combine *S. pneumoniae* isolates against global clones is still unknown due to constraints in the MLVA online database. Therefore, it is advisable to rely on a comprehensive and well-curated MLST database, which has the potential to significantly enhance the exploration of clonal diversity, particularly concerning international clones. Concurrently, MLVA may serve as a complementary method for further elucidating clonal relatedness among isolates, as suggested by Kozáková et al. (36).

Therefore, to assess genetic relatedness and diversity within the collection of the 91 clinical *S. pneumoniae* isolates, phylogenetic-based MLST was employed in combination with MLVA typing. This choice was driven by the long-term evolutionary characteristics offered by MLST, which serve as a baseline for understanding branching patterns, in contrast to the faster evolutionary dynamics of MLVA. This approach allowed us to observe rapid evolutionary changes within the context of the predominant long-term evolutionary lineages provided by MLST. Furthermore, the MLST based phylogenetic tree effectively categorised the MLST CCs into seven respective CCs, namely, MLST CC320, CC9, CC66, CC81, CC90, CC146 and CC311.

Among the CCs identified through MLST analysis, namely, MLST CC320, MLST CC9, and MLST CC66, associations with multiple serotypes have been observed. Specifically, MLST CC66 has demonstrated associations with serotypes 19F, 11A, and 35B. Thus, the isolates of the same CC but with different serotypes were presumed to be capsular switch variants (37). Serotype switching refers to the process by which the loci determining serotypes are transferred between STs, leading to organisms with the same ST expressing more than one capsular type (38). The MLST CC320 clonal lineage was closely related to Taiwan^19F^-14, which shows that ST 236 was originally detected as having the capsular type 19F (39, 40). However, the emergence of serotype 19A in this MLST CC320 suggested that a capsule switching event occurred, involving serotype ST236-19F and serotype 19A strains, which give rise to MLST CC320-19A (41). Serotype 19A shares the same ST designation and allelic profile as serotype 14, suggesting that clinical isolates with comparable genotypic traits but different capsular variations have been discovered within MLST CC9. Moreover, within MLST CC66 associated with serotypes 19F, 11A and 35B share a common allelic profile that groups them into MLVA CC13, although with two distinct MTs. This finding highlights their close genetic relatedness, despite the serotype differences observed. Capsular switching is a prevalent characteristic of pneumococci that is also prevalent before vaccination, driven by factors such as use of antibiotics (37). Therefore, the existence of numerous pneumococcal serotypes and the organism’s ability to switch capsular types have made it difficult to prevent disease through vaccination (42). However, for *S. pneumoniae* to survive, it is imperative that it can swiftly acclimate to clinical interventions and the immunological response of its human hosts (43).

This study also revealed the presence of three unique MLST CCs in the clinical isolates. Specifically, MLST CC66, MLST CC311, and MLST CC146 demonstrated a significant level of clonality across their respective clinical isolates. These clinical isolates were associated with MLVA CC13, MLVA CC3, and MLVA CC6, each characterised by two MTs and two STs. Sjostrom et al. conducted a genetic analysis that clearly showed a pattern: isolates of serotypes with a high potential for invasive illness were genetically closely connected (44). Specifically, serotypes within MLSTs CC9, CC81, CC66, CC311, and CC146 displayed substantial genetic similarity, indicating their elevated invasive disease potential. However, serotype 19F has been included in the group of serotypes that are highly varied and have connections to both invasive disease and a high occurrence among carriers (14). According to both MLVA and MLST analysis, serotype 19F is notably diverse in terms of genetic variation. MLST analysis indicated a substantial degree of diversity within the composition of housekeeping genes among the serotype 19F isolates (45).

Furthermore, nearly all of the MLST CCs (CC320, CC9, CC81, CC66, CC311, and CC146) exhibited a notable tendency for invasiveness, substantiated by the origin of the clinical isolates in this study’s culture collection, which were retrieved from blood samples. This high frequency of invasiveness may be attributed to the fact that the samples originated from inpatients rather than from carriers. Typically, only a small fraction of pneumococci is responsible for causing IPD, whereas the majority of pneumococci inhabit the upper respiratory mucosa of healthy individuals. This is because pneumococcus typically encounters a significant change in its environment, leading to the need for new responses (46). Specifically, MLST CC9 has previously been linked to the England^14^-9 clones, which have been circulating since 1990 in the United Kingdom and have exhibited global dissemination of IPD (14, 40, 47). Nevertheless, documented records have demonstrated that increased rates of IPD due to serotype 19A belong to the rapidly emerging CC320 group, which is related to multidrug-resistant Taiwan^19F^-14 during the initial years following the introduction of PCV7 (48). The invasive disease potential of a strain is primarily determined by variations in its capsular type. However, multiple studies have indicated that genetic background also plays a role in influencing both invasive disease potential and the ability to cause disease (49).

Several serotypes are known to have a strong correlation with clinical and carrier isolates (50, 51). Studies concerning pneumococcal serotypes and associated invasive diseases in 51 nations indicated that serotype 14 was widespread in North America, Latin America, Europe, Asia, Oceania, and Africa (52). Childhood IPD is frequently associated with serotype 19A early in PCV7 vaccination (53, 54). Nevertheless, the implementation of PCV10 and PCV13 resulted in a greater emergence of non-PCV13 serotypes in other countries. Non-PCV13 serotypes have the following descending sequence: 22F, 12F, 33F, 24F, 15C, 15B, 23B, 10A and 38 (54).

However, the introduction of PCV10 into our National Immunisation Programme (NIP) in Malaysia began in late 2020 for children younger than 5 years (55). The clinical isolates were collected in 2017–2019 at various ages before the implementation of the PCV10. However, children younger than five are among the main reservoirs and carriers (56, 57), which may influence the carriage pattern at other ages as well. This may explain the observed high prevalence of PCV10 serotypes in this study. Specifically, the ST236 Taiwan^19F^-14 and ST81 Spain^23F^-1 clones have circulated in Malaysia over the past few decades and continue to persist (58–60). However, PCVs have been available in Malaysia since 2005, mostly through private healthcare channels, for individuals who have financial resources and the ability to obtain the vaccine, despite the ongoing problem of insufficient vaccine coverage (61, 62). Consequently, in regions where pneumococcal vaccines were not incorporated into the NIP, a greater proportion of vaccine serotypes were reported (63). The persistence of specific lineages over long periods raises the question of whether they will survive in the postvaccine era, be replaced by new lineages, or diverge into new subvariants in response to vaccine selective pressure. Thus, the findings from this study are important as baseline data for future serotype surveillance at our setting and other regions, aimed at monitoring potential shifts in serotype distribution and the emergence of novel lineages, especially after the implementation of PCV10 into the NIP. Thus, knowledge of clonal types and the extent of capsule switching is important in understanding or predicting the impact of pneumococcal vaccination since this initiative may drive selection pressure for virulent genotypes to switch capsules and escape coverage by the vaccines (63).

## Conclusion

Overall, several important global pneumococcal lineages have been disseminated over the past few decades and continue to persist until now, as seen in this clinical isolate collection. This study revealed that high virulence clonal isolates are already in circulation. Expanding this analysis to include more hospitals may provide more insight into this pattern.

Moreover, this study revealed a subset of clinical isolates characterised by shared genotypic features but exhibiting variations in capsular variants, implying the possible occurrence of a capsule switching phenomenon. Additionally, it is plausible that this observed pattern may undergo local evolutionary transformations, potentially resulting from serotype switching, thereby leading to the emergence of novel variants. Therefore, a continuous surveillance study is warranted to monitor the dynamics of serotype replacement, particularly in light of the emergence of NVTs. Additionally, two new novel STs, ST18062 and ST18063, were discovered among the clinical isolates in this study. These STs were registered on the PubMLST website and classified as new STs.

The MLVA analysis employed in this study demonstrated its ability to differentiate subgroups within CCs that share the same ST. Additionally, it provides the potential to deduce STs based on the MT. Despite the high discriminatory power of MLVA, its capacity to accurately identify and combine clinical isolates associated with international clones remains uncertain due to limitations within the MLVA online database. Therefore, it is practical to rely on a comprehensive and well-curated MLST database, which offers the potential to significantly enhance the exploration of clonal diversity, particularly in the context of international clones. The adoption of the MLVA typing model to complement MLST is particularly valuable in low-resource settings that lack whole genome sequencing (WGS) and its bioinformatic analytic expertise.

## Supplementary File

Appendix 1 Phylogenetic analysis of the 91 clinical *S. pneumoniae* isolates (labelled M followed by numbers) and 42 reference sequences from the MLST database (labelled REF followed by identity number). ID = strain identification; ST = sequence type; S = singleton. The right column indicates the genetic and demographic background of each isolate according to the site in the phylo-tree.

## Figures and Tables

**Table 1:** A summary of the MLST CCs distribution in relation to their corresponding STs and serotypes, with related Pneumococcal Molecular Epidemiology Network (PMEN) clones

Clonal complex (CC)	Sequence type (ST)	Serotype	PMEN clones	Number of clinical isolates (*n* = 91)
**320**	236	19F	Taiwan^19F^-14	9
	271	19F	SLV of Taiwan^19F^-14	3
	320	19A	DLV of Taiwan^19F^-14	4
		19F		2
	2477	19A	DLV of Taiwan^19F^-14	1
	2648	19F	DLV of Taiwan^19F^-14	1
	4768	19A	TLV of Taiwan^19F^-14	1
	16093	19A	TLV of Taiwan^19F^-14	1
	16499	19F	SLV of Taiwan^19F^-14	1

**9**	9	14	England^14^-9	10
		19A		1
	7	14	SLV of England^14^-9	1

**66**	66	19F	SLV of Tennessee^14^-18	3
		35B		1
	2298	11A	DLV of Tennessee^14^-18	1
		19F		1

**81**	81	6A	Spain^23F^-1	4
	282	6A	SLV of Spain^23F^-1	1
	3604	6A	SLV of Spain^23F^-1	1

**90**	90	6B	Spain^6B^-2	2
	95	6B	SLV of Spain^6B^-2	1
	1661	6B	DLV of Spain^6B^-2	1

**146**	146	6B	-	1
	11811	6B		1

**311**	311	23F	-	2
	**18062**	23F		1

**S**	180	3	Netherlands^3^-31	3
	**18063**	15C	SLV of Greece^21^-30	2
	172	19A	SLV of Colombia^23F^-26	2
	3018	1	SLV of USA^1^-29	2
	36	23F	-	1
	62	11A	-	3
	386	6B	-	2
	440	23F	-	5
	671	14	-	1
	695	19A	-	1
	902	6A	-	1
	1203	6A	-	1
	3781	19A	-	2
	5801	6B	-	1
	5870	6A	-	1
	5872	4	-	1

**S**	6195	23F	-	1
	6208	6A	-	1
	10478	35B	-	1
	12209	23F	-	1
	12400	4	-	1
	16430	19F	-	1

Note: SLV = single locus variant; DLV = double locus variant; TLV = triple locus variant; S = singleton; **Bold** = **new ST**

**Table 2:** The MLST singletons in relation to MLVA CC, ST, MT, serotypes, and demographic

MLST CC	MLVA CC	ID	ST	MT	Serotype	Isolation site	Year	Age
**S**	S	M63	ST282	MT59	6A	Blood	2019	45 years
	S	M60	ST6208	MT70	6A	Eye	2019	17 years
	S	M32	ST12209	MT72	23F	Eye	2018	30 years
	S	M30	ST10478	MT73	35B	Sputum	2018	28 years
	5	M20	ST172	MT32	19A	Blood	2018	18 years
	5	M52	**ST18063**	MT34	15C	Blood	2019	1 month
	5	M54	**ST18063**	MT33	15C	Sputum	2019	4 months
	11	M103	ST440	MT54	23F	Sputum	2017	63 years
	11	M104	ST440	MT54	23F	Sputum	2017	62 years
	11	M8	ST440	MT55	23F	Sputum	2017	26 years
	11	M9	ST440	MT53	23F	Eye	2017	43 years
	11	M36	ST440	MT53	23F	Sputum	2019	29 years
	S	M40	ST5801	MT83	6B	Bronchiole	2019	20 years
	S	M21	ST180	MT71	3	Eye	2018	57 years
	4	M14	ST180	MT29	3	Blood	2018	67 years
	4	M68	ST180	MT30	3	Blood	2019	6 years
	S	M108	ST386	MT64	6B	Blood	2018	48 years
	S	M48	ST386	MT63	6B	Blood	2019	2 years
	S	M35	ST36	MT24	23F	Sputum	2019	51 years
	S	M1	ST5870	MT62	6A	Blood	2017	2 years
	8	M100	ST5872	MT41	4	Blood	2019	66 years
	2	M88	ST695	MT6	19A	Blood	2018	62 years
	S	M27	ST3018	MT1	1	Blood	2018	63 years
	S	M17	ST3018	MT1	1	Sputum	2018	15 years
	S	M19	ST172	MT85	19A	Sputum	2018	62 years
	S	M47	ST6195	MT35	23F	Blood	2019	70 years
	9	M51	ST671	MT44	14	Blood	2019	29 years
	9	M67	ST12400	MT43	4	Sputum	2019	2 years
	10	M55	ST62	MT48	11A	Blood	2019	43 years
	10	M49	ST62	MT49	11A	Sputum	2019	75 years
	10	M3	ST62	MT47	11A	Eye	2017	32 years
	S	M28	ST3781	MT50	19A	Blood	2018	1 year
	S	M102	ST3781	MT50	19A	Sputum	2017	54 years
	S	M89	ST16430	MT52	19F	Blood	2019	7 months
	S	M106	ST902	MT46	6A	Sputum	2018	78 years

Note: MLST CC = MLST clonal complex; MLVA CC = MLVA clonal complex; S = singleton; ID = strain identification; ST = sequence type; MT = MLVA types; **Bold** = **new ST**
