# Supplementary figures and images for: Comparative Genotyping of Malaysian Clinical Isolates of Streptococcus pneumoniae by Multilocus Sequence Typing and Multilocus Variable-Number Tandem Repeat Analysis
Source: Malays J Med Sci. 2025 Feb 28;32(1):69–87. doi: 10.21315/mjms-09-2024-677 (PMC12097172; doi:10.21315/mjms-09-2024-677)

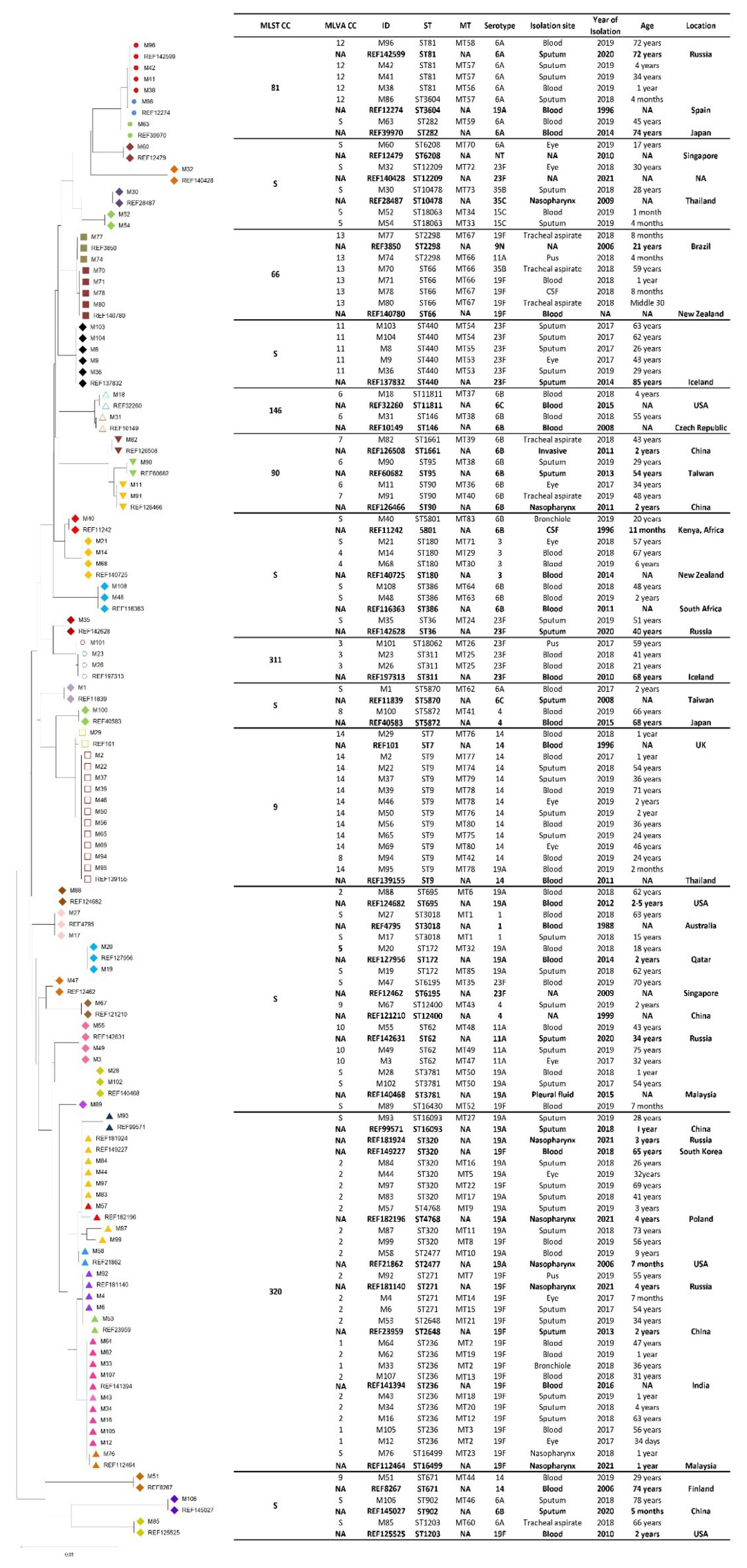

Supplement: Appendix 1 — Phylogenetic analysis of the 91 clinical S. pneumoniae isolates (labelled M followed by numbers) and 42 reference sequences from the MLST database (labelled REF followed by identity number). ID = strain identification; ST = sequence type; S = singleton. The right column indicates the genetic and demographic background of each isolate according to the site in the phylo-tree. [file 07mjms3201_oaf4.tif]
